# A Phase II Trial of Geriatric Assessment‐Guided Selection of Treatment Intensity in Older Adults With AML

**DOI:** 10.1002/ajh.27694

**Published:** 2025-04-29

**Authors:** Vijaya R. Bhatt, Christopher S. Wichman, Thuy T. Koll, Alfred L. Fisher, Tanya M. Wildes, Michael Haddadin, Ann M. Berger, James O. Armitage, Sarah A. Holstein, Lori J. Maness, Krishna Gundabolu

**Affiliations:** ^1^ Division of Hematology‐Oncology, Department of Internal Medicine University of Nebraska Medical Center Omaha Nebraska USA; ^2^ Fred & Pamela Buffett Cancer Center University of Nebraska Medical Center Omaha Nebraska USA; ^3^ Department of Biostatistics University of Nebraska Medical Center Omaha Nebraska USA; ^4^ Division of Geriatrics, Gerontology, and Palliative Medicine, Department of Internal Medicine University of Nebraska Medical Center Omaha Nebraska USA; ^5^ College of Nursing ‐ Omaha Division University of Nebraska Medical Center Omaha Nebraska USA

**Keywords:** acute myeloid leukemia, geriatric assessment, mortality, older adults, precision oncology

## Abstract

How to select the appropriate intensity of chemotherapy in older adults with acute myeloid leukemia (AML) remains an unanswered question. In a phase II trial of older adults ≥ 60 years with AML (*n* = 73), we used geriatric assessment (measures of comorbidity burden, physical and cognitive function) to determine fitness for intensive chemotherapy. We integrated the geriatric assessment and genetic test results to personalize the selection of chemotherapy intensity with a goal to reduce early mortality (NCT03226418). Broad eligibility criteria allowed enrolling patients representative of those treated in real‐world practices: 45% of patients were ≥ 70 years, 57% had ≥ 2 comorbidities, 27% had a history of solid malignancies, and 74% had impairments in ≥ 2 geriatric assessment domains used to assign treatment intensity. Thirty‐two percent of patients resided in rural areas, and 45% were comanaged with community oncologists. The median time from enrollment to therapy initiation was 1 day (range 0–13). Eight patients (11%) received intensive chemotherapy; others received low‐intensity chemotherapy. Mortality at 30 days from diagnosis was 6.8% (95% confidence interval, CI 3.0%–15.1%) and at 90 days was 21.9% (95% CI 14.0%–32.7%). One‐year survival was 45.9% (95% CI 35.6%–59.3%). Our study demonstrates that pre‐treatment geriatric assessment in older adults with AML is feasible, can identify several functional impairments, and guide the selection of treatment intensity. A randomized trial is necessary to confirm the survival benefit of this approach over the traditional approach of treatment selection.

**Trial Registration:** NCT03226418

## Graphical abstract

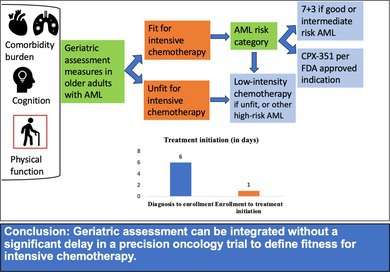

## 1 Introduction

How to select appropriate intensity of chemotherapy in older adults with acute myeloid leukemia (AML) remains an unanswered question. While consensus‐driven subjective criteria exist, prospectively validated objective criteria to determine fitness for intensive chemotherapy are lacking [1, 2]. Geriatric assessment can capture multiple domains of health and functioning in older adults with AML and can identify impairments even in patients considered to have excellent performance status [3]. In older adults with solid malignancies as well as in AML, measures of geriatric assessment can predict the risk of chemotherapy toxicities [1, 4, 5]. In AML, several studies have demonstrated that functional impairments, identified by geriatric assessment, are associated with higher risk of mortality [1, 6, 7, 8, 9, 10]. In particular, the presence of higher comorbidity burden (hematopoietic cell transplant comorbidity index or HCT CI ≥ 3) or impairments in physical function or cognitive function at the time of diagnosis of AML increase the risk of mortality [4, 6, 9, 11]. For example, a study from MD Anderson Cancer Center highlighted a high early mortality rate of 29% and a median survival of 19 weeks with intensive chemotherapy in older adults with AML who had a HCT CI score of ≥ 3 [9]. Klepin et al. in one of the first geriatric assessment studies in AML, demonstrated an increased risk of mortality following intensive chemotherapy in older adults with impairment in cognitive function (hazard ratio of 2.5) or physical function (hazard ratio of 2.2) [6]. Thus, older adults with HCT CI ≥ 3 or impairment in physical or cognitive function, who receive intensive chemotherapy, are at a higher risk of mortality.

The role of leukemia biology is well established in older adults with AML treated with intensive chemotherapy, with differing survival based on AML risk categories [12]. Oncologists may be concerned about the feasibility of waiting for results of genetic testing when patients present at diagnosis. However, several studies have demonstrated the safety of awaiting the results of appropriate genetic testing before treatment initiation in stable older adults with AML, including in the era of venetoclax and other novel treatment‐based combinations [13, 14, 15, 16, 17]. Thus, integrating both geriatric assessment and genetic profiling of leukemic cells could provide an innovative strategy to answer the questions of determining fitness for intensive chemotherapy and selection of appropriate intensity of chemotherapy in older adults with AML.

Our study hypothesis was that the use of geriatric assessment and genetic profiling to personalize therapy selection will result in lower rates of early mortality in older adults with AML. We designed a Phase II trial to test this hypothesis. Many adults are diagnosed with AML unexpectedly; patients, particularly those with multimorbidity and multiple functional impairments, face a high risk of early mortality as high as 20%–40% [18, 19, 20]. Reducing early mortality during induction is crucial to improve the chance of proceeding to curative therapy such as allogeneic stem cell transplantation. Reducing early mortality is also vital to ensure time for patients and their families to engage in end‐of‐life planning when facing a life‐threatening diagnosis. These issues highlight the importance of this study aimed at using a new treatment selection strategy to reduce early mortality. We have previously published results of a pre‐planned interim analysis [21], and in this report, we present the results of the fully accrued trial.

## 2 Methods

### 2.1 Patient Eligibility

Eligibility criteria were broad to increase representation of older adults with AML, who are likely to be treated in real‐world clinical practices. Inclusion criteria included adults ≥ 60 years with a new diagnosis of AML (other than acute promyelocytic leukemia) or high‐grade treatment‐related myeloid neoplasm. Patients with AML equivalents such as myeloid sarcoma were allowed to participate. Other inclusion criteria included Karnofsky Performance Status (KPS) ≥ 60. The study permitted use of cytoreductive therapy or leukapheresis for hyperleukocytosis, as well as chemotherapy for other concurrent malignancies. Older adults with comorbidities or organ dysfunction, such as ejection fraction < 45%, creatinine of ≥ 2 mg/dL, or bilirubin ≥ 2 times upper limit of normal, were permitted to participate and receive low‐intensity chemotherapy. Key exclusion criteria included uncontrolled systemic infection, clinically significant arrhythmia, myocardial ischemia, or active congestive heart failure within the past 2 weeks.

### 2.2 Geriatric Assessment and Fitness Determination

Trained research nurses completed a battery of geriatric assessments prior to treatment initiation. These included HCT CI (measure of comorbidity burden), Katz Activities of Daily Living (ADL) Index, and Lawton Instrumental ADL (IADL) Index (self‐reported measures of physical function), Short Physical Performance Battery (SPPB) (objective measure of physical function), and Montreal Cognitive Assessment (MoCA) (measure of cognitive function). Based on prior studies in AML demonstrating an association between the risk of mortality and impairments in any of these geriatric domains [4, 6, 9, 11], patients were deemed fit for intensive chemotherapy if they scored well on all of these measures. The cutoffs used to determine impairment in these geriatric assessment measures are based on previously well‐established criteria for various measures (Table 1) [9, 22, 23, 24, 25]. We used a HCT CI of ≥ 3 as one of the criteria to assign low‐intensity chemotherapy based on the higher risk of early mortality with intensive chemotherapy in this population [9]; however, with the approval of CPX351 for treatment‐related AML in August 2017, we made a protocol amendment. For patients with treatment‐related AML (who by virtue of prior cancer treatment would be categorized as having an HCT CI score of 3), we required a HCT CI of ≥ 5 before avoiding use of CPX351, which had shown survival benefit in a Phase 3 trial conducted specifically in therapy‐related AML [26].

**TABLE 1:** Definition of Fit and Unfit status according to the geriatric assessment.

Geriatric domains	Fit: all criteria (scores)	Unfit: one or more criteria (scores)	Number and percentage of patients meeting threshold for vulnerable	
Comorbidity burden				
Hematopoietic Cell Transplantation Comorbidity Index (HCT CI)	0‐2^a^	3 or more	40	55%
Physical function				
Katz Activities of Daily Living (ADL) Index	6	5 or less	21	29%
Lawton Instrumental ADL (IADL) Index	8	7 or less	16	22%
Short physical performance battery (SPPB)	10–12	9 or less	51	70%
Cognitive function				
Montreal cognitive assessment (MoCA)	26–30	25 or less	47	64%

*Note*: The number indicates scores of different tests.

^a^ Patients with therapy‐related AML needed ≥ 2 scores in addition to the history of prior malignancy to be considered unfit. This modification was made to allow using CPX 351.

Other assessments, not used for treatment assignment, included Patient Health Questionnaire‐9 (PHQ‐9) (depression screen) [27], Mini Nutritional Assessment‐Short Form (MNA) (nutritional screen) [28, 29], and Medical Outcomes Study Social Function Scale (measure of social support) [30]. These additional assessments were used to capture the baseline characteristics of patients but were not used for treatment assignment because of less robust associations with mortality [1]. Depression was already captured in HCT CI; hence the results of the depression screen were not used for treatment assignment.

### 2.3 Study Design and Treatment Selection

**FIGURE 1:**
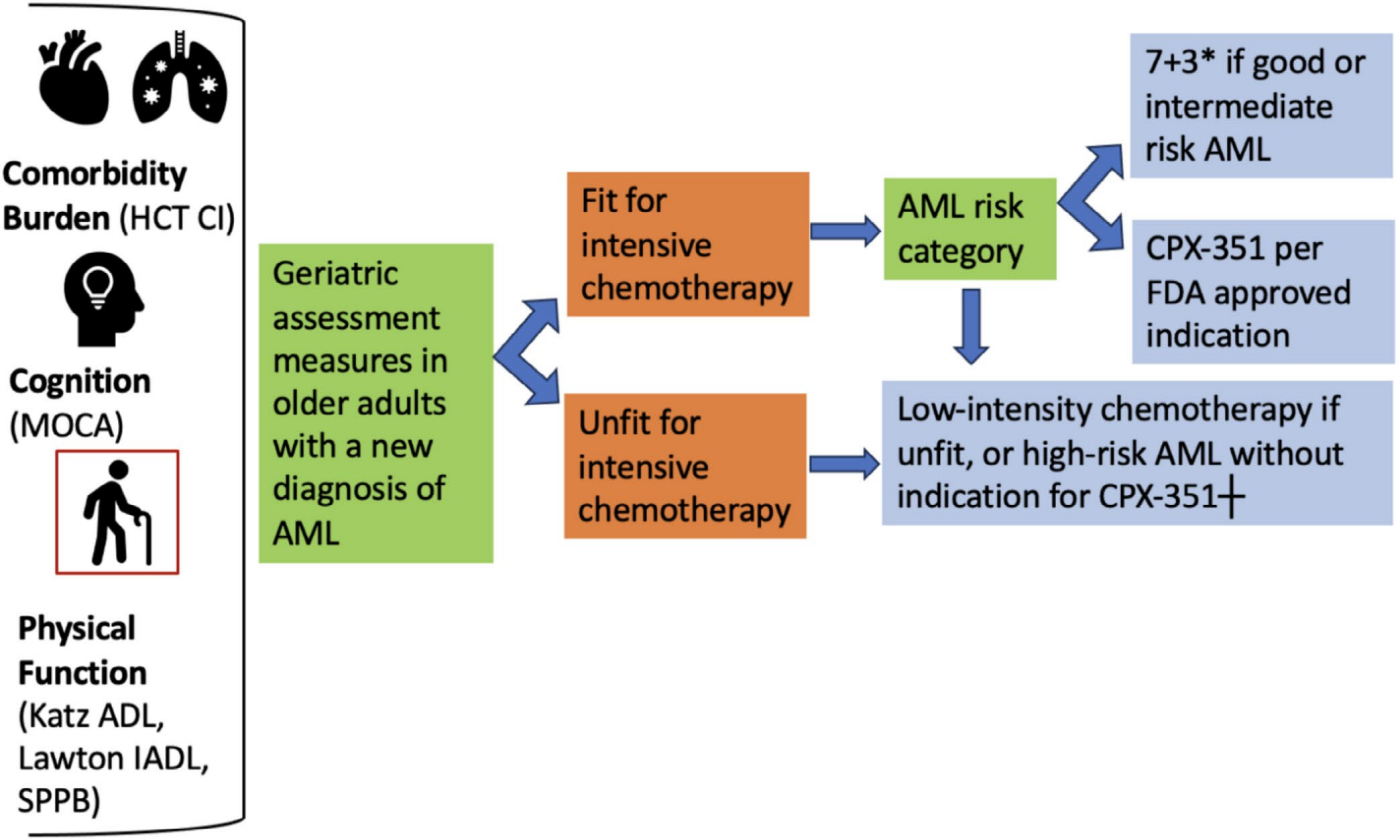
Personalized therapy selection. ADL; activities of daily living, HCT CI; Hematopoietic Cell Transplantation Comorbidity Index, IADL; instrumental ADL, MoCA; montreal cognitive assessment, SPPB; short physical performance battery. *Allowed to add gemtuzumab ozogamicin or midostaurin as clinically indicated. Low‐intensity chemotherapy included a hypomethylating agent or low‐dose cytarabine with or without a novel drug. Upon accelerated approval of venetoclax, the venetoclax‐based combination was the preferred option. Consolidation chemotherapy included intermediate‐dose cytarabine, CPX‐351, or continuation of low‐intensity chemotherapy. [Color figure can be viewed at wileyonlinelibrary.com]

This single‐center phase II trial used the results of geriatric assessment and genetic profiling to select the intensity of treatment (Figure 1). Genetic profiling was based on conventional karyotyping and fluorescence in situ hybridization (FISH); the risk categories followed the 2017 European LeukemiaNet (ELN) criteria [31]. The study used standard‐of‐care mutation testing, which required insurance approval and had an anticipated turnaround time of 1–2 weeks. Therefore, this pragmatic study used any available mutation test results to risk stratify AML into various categories but did not mandate waiting for multigene mutation panel results prior to treatment selection.

Patients, who were considered fit by geriatric assessment, and had either good or intermediate‐risk AML, received intensive chemotherapy, such as a combination of anthracycline and cytarabine (7 + 3). Gemtuzumab or midostaurin could be added to intensive chemotherapy, respectively for good‐ or intermediate‐risk AML, or for FLT3 mutated AML. Fit patients, who had treatment‐related AML or AML with myelodysplasia‐related changes, received liposomal daunorubicin‐cytarabine (CPX 351) in accordance with the FDA‐approved indication for CPX 351. Patients who were not considered fit by geriatric assessment or those with high‐risk AML not meeting the FDA‐approved indication for CPX 351 received low‐intensity chemotherapy, such as a hypomethylating agent with or without novel drugs. Upon accelerated approval of venetoclax in November 2018, venetoclax‐based combination was the preferred low‐intensity chemotherapy option. In older adults with adverse‐risk AML, intensive chemotherapy results in response rates that can be similar to those achieved with lower‐intensity chemotherapy [32], and getting these patients into remission with low‐intensity chemotherapy could preserve their fitness for allogeneic stem cell transplantation.

Safety monitoring and disease restaging followed standard‐of‐care practices. The study allowed patients to receive these standard‐of‐care chemotherapy regimens and restaging bone marrow biopsies to be performed in community cancer centers outside of our center. Survival data were collected for up to 2 years.

The study was registered with ClinicalTrials.gov identifier (ClinicalTrials.gov identifier: NCT03226418). The institutional review board at the University of Nebraska Medical Center approved the study. The trial was conducted in accordance with the principles of the Declaration of Helsinki. All participants provided written informed consent.

### 2.4 Study End Points

The primary endpoints of the study were 90‐day mortality and complete remission (as best response). Mortality from any cause within the first 90 days from the time of diagnosis counted towards 90‐day mortality. Response criteria were based on the definitions provided by the 2003 International Working Group [33]. Secondary endpoints included impairments detected by geriatric assessment, receipt of allogeneic stem cell transplant, and overall survival. Overall survival was defined as the time from the date of diagnosis to the date of death due to any cause.

### 2.5 Statistical Analyses

#### 2.5.1 Sample Size Justification

An optimal Simon two‐stage design [34] was used to test the null hypothesis that 60% versus the alternative of 75% will be alive at 3 months (benchmark of 60% survival or 40% mortality at 90 days was based on a prior published institutional data [35]). A sample size of 67 patients achieves 80% power to detect a difference of 15% at a significance level of 0.05. Accounting for an attrition rate of 10%, a total of 75 patients would be enrolled. PASS 11 software [36] was used to conduct all sample size analyses.

#### 2.5.2 Data Analysis

Data were descriptively summarized using median scores, ranges, and proportions to describe baseline characteristics of the study population. The proportion of patients with impairments across various domains of geriatric assessment are presented. We calculated the median time in days from diagnosis to trial enrollment and to therapy initiation. The proportion of patients who underwent allogeneic stem cell transplant is presented. The method of inversion [37] was used to generate an interval estimate for the proportion of 90‐day mortality in an intent‐to‐treat population. The Kaplan–Meier method was used to estimate the overall survival (OS) distribution. If a patient was lost to follow‐up, survival was censored at the date of last contact.

## 3 Results

### 3.1 Baseline Characteristics and AML Risk Categories

We consented 75 patients between July 2017 and October 2022; 2 patients were determined not to be eligible because of the presence of uncontrolled cardiac arrhythmia and lack of AML diagnosis (Figure S1). A retrospective review of the institutional cancer registry showed 202 older adults with a new diagnosis of AML during the study period, which highlights that one‐third of the patients were enrolled in our study (data not available to identify reasons why other patients were not enrolled). Baseline characteristics of the trial participants (*n* = 73) included a median age of 69 years (range 60–87 years) with 45% of patients being 70 years or older, 49% female, 92% white, and a median KPS of 80 (range 60–100). Median white blood count (WBC) on presentation was 5.5 × 10^9^/L (range 1.7–128. 2 × 10^9^/L; 8 patients had WBC > 50 × 10^9^/L), and median bone marrow blast was 45% (range 3%–93%).

Based on the 2016 World Health Organization classification [38], patients had therapy‐related myeloid neoplasms (22.0%), AML with mutated NPM1 (19.2%), AML with myelodysplasia‐related changes (17.8%), AML with mutated RUNX1 (17.8%), or AML not otherwise specified (15.1%); other categories included core‐binding factor AML (2.7%), AML with biallelic CEBPA mutation (2.7%), or myeloid sarcoma (2.7%) (Table S1). Risk categories included adverse (60%), intermediate (22%), and good‐risk AML (18%). Among fit patients, 62.5% had adverse‐risk and 37.5% had intermediate‐risk AML, whereas among unfit patients, 61.5% had adverse‐risk, 20% had intermediate‐risk, and 18.5% had good‐risk AML. Among the patients with good‐risk AML (*n* = 13), most were categorized as good‐risk based on NPM1 or biallelic CEBPA mutation; two patients had core‐binding factor AML (Table S2). The most common mutations included TET2, NPM1, RUNX1, ASXL1, TP53, SRSF2, IDH2, DNMT3A, NRAS, FLT3 ITD, IDH1, and STAG2 mutations (Figure S2). Based on zip code data, 23 patients (32%) resided in rural areas, and 50 patients (68%) resided in urban areas (Figure S3a). Thirty‐three patients (45%) were comanaged with community oncologists, and 15 (21%) received part of their chemotherapy in community cancer centers (Figure S3b).

### 3.2 Results of Geriatric Assessment

**FIGURE 2:**
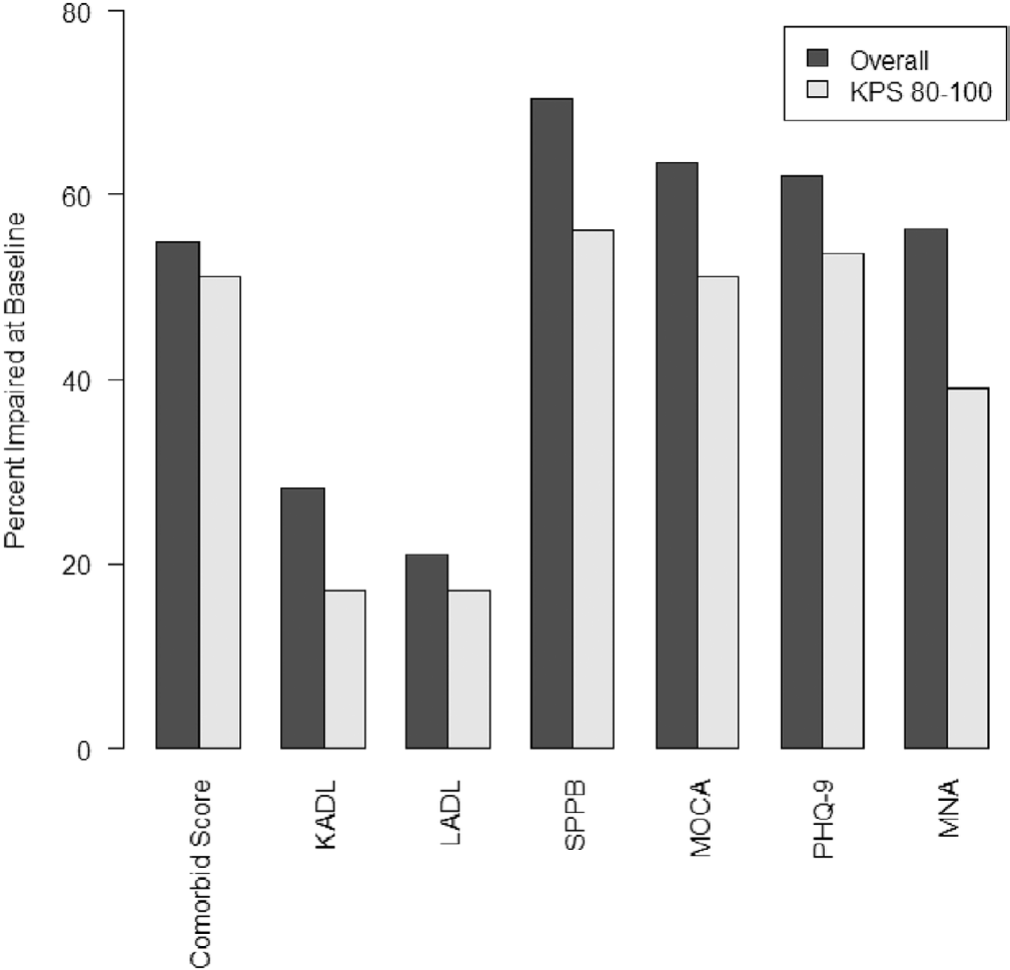
Impairments in geriatric assessment results based on performance status. KADL: Katz Activities of Daily Living (ADL) Index, LADL: Lawton Instrumental ADL Index, MOCA: montreal cognitive assessment, MNA: mini nutritional assessment‐short form, PHQ‐9: Patient Health Questionnaire‐9, SPPB: short physical performance battery.

The majority of patients had HCT CI of ≥ 3 (55%), impairment in objective physical function, as measured with the short physical performance battery (70% had a score of 9 or less), or cognitive impairment as measured by the Montreal Cognitive Assessment (64% had a score of 25 or less). Self‐reported ADL and IADL were impaired in 29% and 22%, respectively. Depression screen (PHQ‐9 score of ≥ 10) and nutritional screen (MNA score of ≤ 11) were abnormal in 33% and 56% at baseline. Impairments were also common in patients with KPS score of 80–100 (Figure 2). The most common comorbidities included depression or anxiety (28.8%), prior solid malignancy (27.4%), diabetes (24.7%), cardiovascular disease (24.7%), and obesity (19.2%) (Table S3). A total of 42.4% had 0–1 comorbid conditions, whereas 57.6% had 2 or more comorbid conditions. Among the patients with a history of prior solid malignancy (*n* = 20), 7 patients had finished chemotherapy within the past 2 years, and 4 patients were receiving hormonal therapy or monoclonal antibody up until the time of diagnosis of AML. Additionally, 10.9% of patients had ejection fraction ≤ 50%, 6.8% had creatinine of > 2 mg/dL, and 6.8% had a diagnosis of cirrhosis.

**FIGURE 3:**
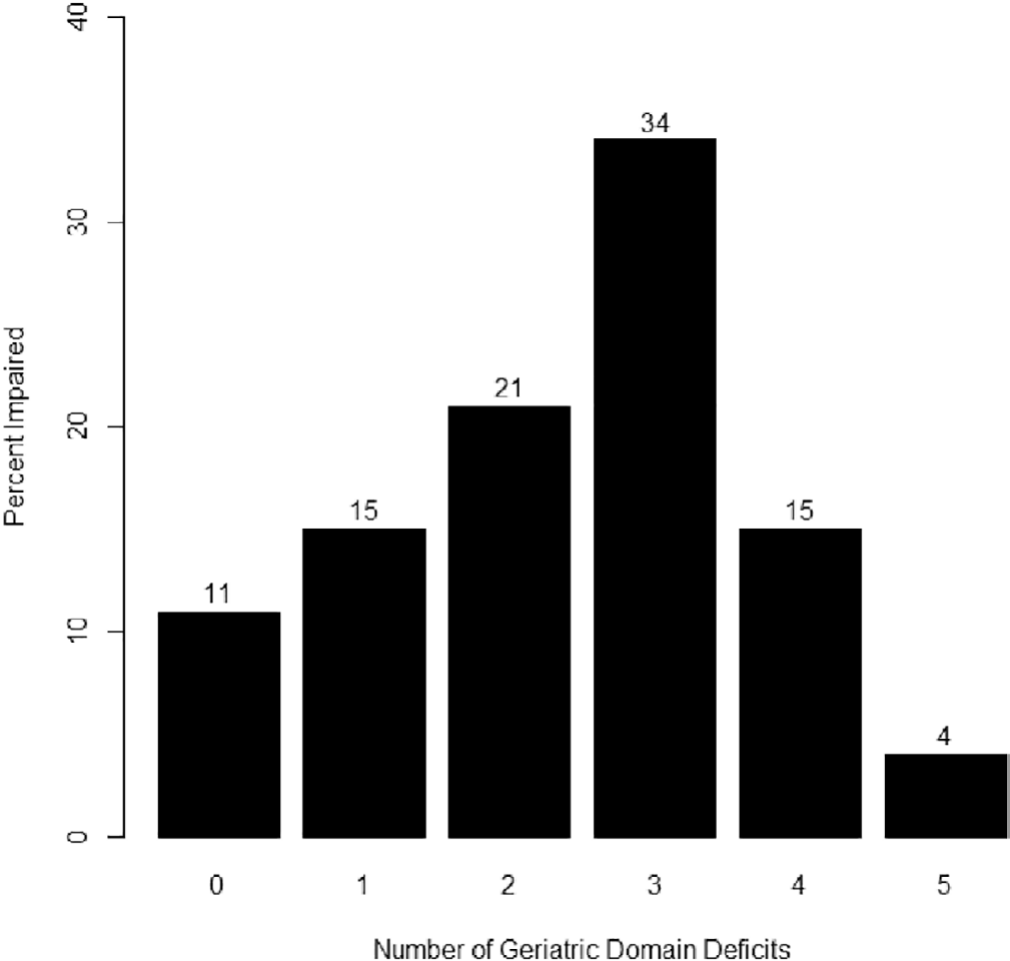
Frequency of impairments in geriatric assessment domains (among the domains used to determine fitness for intensive chemotherapy).

Among the domains used to determine fitness for intensive chemotherapy, 74% of patients had impairment in more than one of these geriatric assessment domains (Figure 3, Table S4). Eight patients were deemed to be fit for intensive chemotherapy based on the baseline geriatric assessment results (Table S5). Whereas the median ADL and IADL scores were similar between fit and unfit groups, the two groups appeared to differ based on the comorbidity burden and the results of objective physical function and cognitive function testing.

### 3.3 Treatment Selection and Initiation

**FIGURE 4:**
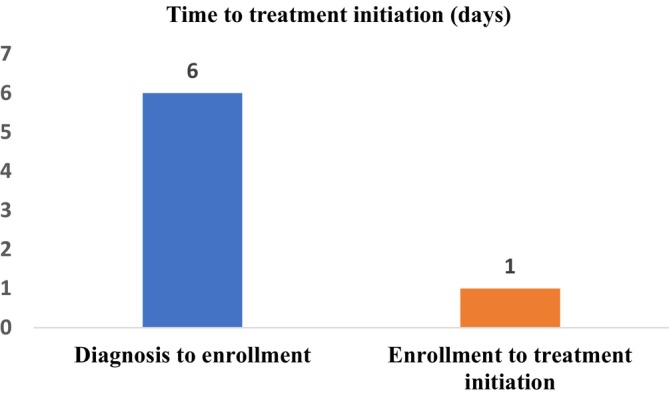
Time to treatment initiation from diagnosis and enrollment. [Color figure can be viewed at wileyonlinelibrary.com]

The selection of treatment intensity followed the trial protocol in all patients. Based on the protocol, eight patients (11%) received intensive chemotherapy: CPX 351 (*n* = 4) or 7 + 3 based regimens (*n* = 4). All 4 patients who received CPX351 had a diagnosis of AML with myelodysplasia‐related changes and HCT CI of 0–1. Other patients received low‐intensity chemotherapy: azacitidine or decitabine and venetoclax (*n* = 43, 59%), decitabine or azacitidine alone prior to approval of venetoclax (*n* = 18, 24%), and other (*n* = 4, 6%). All 13 patients with good‐risk AML, with one exception, had 2 or more impairments and received low‐intensity chemotherapy (Table S2). The median time from diagnosis to therapy initiation was 6 days (range 0–77 days) and from enrollment to therapy initiation was 1 day (range 0–13) (Figure 4). The patient who waited for therapy initiation for 77 days from diagnosis had treatment‐related myeloid neoplasm and was referred to us after a significant delay outside of our center. The patient who waited for therapy initiation for 13 days had treatment at a community cancer center, and the delay was related to a delay in scheduling chemotherapy. A total of 30 patients (*n* = 41.1%) underwent allogeneic stem cell transplantation. Among fit patients, 75% had an allogeneic stem cell transplant, whereas 37% of unfit patients underwent transplant.

### 3.4 Patient Outcomes

Complete remission (CR) (40%) or CR with incomplete count recovery (12%) as the best responses to study treatment was noted in 52% of patients; other responses included morphological leukemia‐free state in 2.8%, resistant disease in 35.6%, and non‐evaluable in 9.6%. The chances of CR/CRi were 69.2%, 56.3%, and 45.5% for patients with good, intermediate, and adverse‐risk AML, respectively (Table S6). The chances of CR/CRi were 50% and 52.3% for fit patients (treated with intensive chemotherapy) and unfit patients, respectively.

**FIGURE 5:**
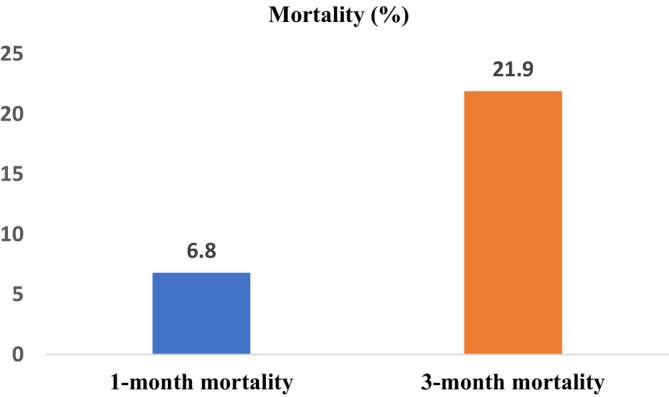
One‐month and three‐month mortality among the study participants. In an intent‐to‐treat population, 5 out of 73 and 16 out of 73 participants died by 1 month and 3 months, respectively. [Color figure can be viewed at wileyonlinelibrary.com]

With a median follow‐up of 12 months, mortality at 30 days from diagnosis was 6.8% (95% confidence interval, CI 3.0%–15.1%) and at 90 days was 21.9% (95% CI 14.0%–32.7%) (Figure 5), thus the study met its primary endpoint. One‐year Kaplan–Meier estimated OS was 45.9% (95% CI 35.6%–59.3%) (Figure S4a). For patients with good‐risk AML, mortality at 30 and 90 days from diagnosis was 0% and 7.7%. Of 13 patients with good‐risk AML, 8 and 6 patients lived for 1 year and 2 years, respectively. Mortality at 30 days was 0% versus 7.7%, and 90‐day mortality was 12.5% versus 23.1% for fit versus unfit patients, respectively. One‐year Kaplan–Meier estimated OS was 42.9% versus 46.3% for fit versus unfit patients, respectively (Figure S4b,c). The cause of death was AML in 40% of fit patients and 51% of unfit patients (Table S7).

## 4 Discussion

Our study demonstrates that pre‐treatment geriatric assessment is feasible in AML, can identify impairments even in patients with excellent performance status, can guide treatment selection, and may have the potential to reduce early mortality, particularly one‐month mortality, in older adults with multimorbidity and multiple functional impairments. Our pragmatic trial has innovative research design components and novel findings that can advance the precision oncology approach in older adults with AML. Research design innovations include an objective model to define fitness for intensive chemotherapy and use of geriatric assessment and genetic profiling to personalize selection of treatment intensity. These strategies aimed to address the unanswered question of personalizing selection of intensive versus low‐intensity chemotherapy in older adults with AML. We also designed a study with broad eligibility criteria (e.g., allowing patients with organ dysfunction and concurrent solid malignancy) and allowed co‐management of patients (e.g., treatment initiation, disease restaging, and adverse event management) with community oncologists to ensure inclusion and representation of older adults treated in real‐world practices.

Several factors highlight the success of research design and inclusion strategies. First, we enrolled one‐third of all older adults with newly diagnosed AML managed by our center during the study period. This exceeds the rate of trial enrollment (7.1%) among adults with cancer in the United States [39], and highlights that an older adult specific study can address a concern of lower rate of representation of older adults in cancer trials [40]. Second, the study participants represent older adults with AML treated in real‐world practices: 45% of patients were 70 years or older, 57% had ≥ 2 comorbidities, and 27% had a history of prior solid malignancies. Some of the patients in our study also had comorbidities such as creatinine of > 2 mg/dL or cirrhosis. Such patients with multimorbidities, functional impairments, and organ dysfunction are routinely encountered in clinical practices but are often excluded from participating in clinical trials [12, 41, 42, 43]. Third, a total of 32% of patients resided in rural areas, and 45% were comanaged with community oncologists, including 21% who received part of chemotherapy in community cancer centers. In consultation with the study investigators, community oncologists were also involved in comanaging adverse events and disease restaging at community cancer centers. Such academic‐community collaboration can reduce barriers to participation in clinical trials and improve access to trials for patients with AML [44]; of whom one‐half are managed in community cancer centers in US [18]. Such a comanagement approach is particularly feasible in trials that use standard‐of‐care chemotherapy and can support patient preferences to be treated closer to home.

Novel findings of this study include demonstration of the feasibility of geriatric assessment‐guided treatment selection in AML. Feasibility is highlighted by the fact that baseline geriatric assessments were completed in all study participants prior to treatment initiation. Timeliness of completing geriatric assessment is demonstrated by a short median time from diagnosis to enrollment (6 days) and enrollment to treatment initiation (1 day). Consistent with prior studies, we also demonstrated high rates of impairments in geriatric assessments even among patients who were felt to have an excellent performance status (KPS of 80–100), highlighting the value of performing geriatric assessments over capturing performance status alone [3].

Early mortality increases steeply with increasing comorbidity burden [20], and reducing early mortality in older adults with multimorbidity and multiple functional impairments requires innovative strategies. While the early mortality rate met the study endpoint, because of the biases associated with the comparison to the historical data [35], a randomized trial is necessary to confirm whether integrating geriatric assessment and genetic test to select treatment intensity improves survival over the traditional approach. Nonetheless, one‐month mortality (6.8%) rate was low, which may be because of avoidance of intensive chemotherapy in unfit patients at high risk of fatal complications. Among 8 patients who were fit and received intensive chemotherapy, mortality at 30 days and 90 days from diagnosis was 0% and 12.5%, which appeared lower than 7.7% and 23.1% noted in 65 unfit patients. This finding supports that measures of geriatric assessment can identify patients who can tolerate intensive chemotherapy. Geriatric assessment results were shared with the oncologists and were often used to guide supportive care interventions, which can improve outcomes.

Many patients received low‐intensity chemotherapy but were able to subsequently undergo allogeneic stem cell transplantation. The presence of high rates of impairments because of broad eligibility criteria and stringent definitions for fitness for intensive chemotherapy likely explains a low proportion of patients receiving intensive chemotherapy. Several patients with good‐risk AML received low‐intensity chemotherapy; however, all 13 patients with good‐risk AML, with one exception, had 2 or more impairments. In this population, early mortality was very low at 0% and 7.7% at 30 and 90 days from diagnosis; six out of 13 patients with good‐risk AML lived for 2 years or beyond. These results compare favorably to older adults with good‐risk AML based on NPM1 mutations treated with intensive chemotherapy [45], which constituted the majority of good‐risk patients in our study. A total of 41% of patients underwent allogeneic stem cell transplantation, which is comparable to or even higher than prior studies in older adults including those treated with intensive chemotherapy [12, 26]. This raises the possibility of whether personalized treatment selection can better maintain the function of patients, allowing them to be candidates for allogeneic stem cell transplantation. This is supported by our previously published exploratory findings that about three‐quarters of patients in this trial maintained or improved their cognitive and physical function at 3 months post‐treatment, as compared to baseline [46].

The study has several strengths, including components of innovative research design and novel findings discussed previously. This is, to our knowledge, the first study to utilize geriatric assessment and genetic profiling to guide treatment selection in older adults with AML. The study findings are expected to advance a precision oncology approach in the population and serve as a platform for future precision oncology trials [47].

The potential limitations of the study include a modestly sized, non‐randomized trial and lack of routine use of multigene mutation panel testing. Future trials should use rapid multigene mutation panel test results (such as those being used in cooperative group trials [48]) in determining leukemia biology and selecting treatment intensity, which was not routinely available in this resource‐constrained pragmatic study. The presence of specific gene mutations can now qualify as AML with myelodysplasia‐related changes [49]; specific mutations may also identify patients who have a survival benefit from CPX 351 over 7 + 3 [50]. Several targeted agents are now approved or in clinical trials for subsets of AML with specific mutations or genetic changes (e.g., IDH1, IDH2, FLT3 ITD, FLT3 TKD, NPM1 mutations or KMT2A rearrangement), highlighting the importance of incorporating multigene mutation panel testing in precision oncology trials [48].Additionally, TP53 mutated AML is associated with dismal outcomes, which may not improve with the addition of drugs such as venetoclax, thus requiring a separate clinical trial when possible [51]. The study did not use the recent European LeukemiaNet 2024 genetic risk classification that can differentiate outcomes more accurately in older adults treated with venetoclax‐based regimens and should be incorporated in future studies [51, 52].

The study was not designed or powered to compare the outcomes of fit versus unfit patients. Numerically, fit versus unfit patients were less likely to have good‐risk AML (0% vs. 18.5%), less likely to have early mortality (0% vs. 7.7% at 30 days), and more likely to undergo transplant (75% vs. 37%); remission rates and OS were similar. The cause of death was AML in 40% of fit patients and 51% of unfit patients. Two deaths post‐transplant in fit patients accounted for 40% mortality in this population. The most common cause of death in unfit patients was AML, which could add to the debate of whether more intensive chemotherapy versus newer lower‐intensity combination chemotherapy could improve the outcomes of this population [53, 54]. However, the OS of unfit patients was comparable to that of fit patients, which also indicates a possibility that our approach to treatment assignment may have improved the OS of unfit patients.

Future larger studies will be necessary to determine optimal treatment for patients with different levels of fitness. While significant controversy exists regarding whether older adults benefit from intensive versus low‐intensity chemotherapy [53, 54], a recent randomized trial in younger adults (18–59 years) has demonstrated that the outcomes of venetoclax‐decitabine induction are non‐inferior to those associated with intensive chemotherapy, with results appearing in favor of venetoclax‐decitabine induction for those aged between 40 and 59 years, and those with adverse‐risk AML, but not for those with RUNX1::RUNX1T1 fusion [32]. The feasibility of incorporating geriatric assessment, as shown in our study, can further enhance a precision oncology trial design in older adults with AML, and with a randomized controlled trial, answer the question of what subset of older adults with AML benefit from intensive versus low‐intensity chemotherapy. Future trials should also incorporate patients' preferences, and patient‐reported outcomes associated with intensive versus low‐intensity chemotherapy [55, 56, 57, 58]. Such data can guide selection of treatment that is most likely to achieve patients' goals of care and be aligned with patients' preferences.

In conclusion, we have demonstrated the feasibility and promising results of an approach to integrate geriatric assessment and genetic profiling to guide treatment intensity in older adults with AML. While geriatric assessment results can provide an objective model to determine fitness for intensive chemotherapy, a randomized trial is necessary to confirm whether integrating geriatric assessment and genetic tests to select treatment intensity improves survival over the traditional approach. Our study results demonstrate the success of broad eligibility criteria and academic‐community collaboration in expanding access to clinical trials and in enrolling older adults who have multimorbidity or reside in rural areas. With the ongoing global crisis of cancer in an aging population, as well as challenges associated with access to trials in rural areas, innovation is essential in research design and implementation. In this context, our approach to personalize selection of treatment intensity in older adults with AML, and our research design considerations including broad eligibility criteria and collaboration with community oncology centers can serve as a model for future trials that aim to answer issues specific to treatment selection in older adults with AML or other hematologic malignancies.

## Author Contributions

V.R.B., C.S.W., T.T.K., A.M.B., J.O.A., S.A.H., K.G., and L.J.M. conceived and designed the study. V.R.B., K.G., and L.J.M. enrolled patients in the clinical trial, C.S.W. performed statistical analysis, all authors contributed to data interpretation, participated in manuscript writing, approved the manuscript for publication, and are accountable for all aspects of the work.

## Ethics Statement

The study was approved by the institutional review board at the University of Nebraska Medical Center and was conducted according to the Declaration of Helsinki.

## Consent

All study participants signed a written consent to participate in the study.

## Conflicts of Interest

V.R.B. reports participating in the Safety Monitoring Committee for Protagonist, serving as a member of the National Comprehensive Cancer Network Acute Myeloid Leukemia Panel, as an Associate Editor for the journal, Current Problems in Cancer, and as a contributor for *BMJ Best Practice*, and receiving consulting fees from Imugene, Sanofi, Taiho, research funding (institutional) from Cynata Therapeutics, MEI Pharma, Actinium Pharmaceutical, Sanofi U.S. Services, Abbvie, Pfizer, Incyte, Jazz, and National Marrow Donor Program, and drug support (institutional) from Chimerix for a trial. T.M.W. reports consulting for Pfizer, Sanofi, and Janssen. J.O.A. reports serving as an Editor for ASCO Post, Consultant & Scientific Advisory Board member for Syncromune, and Consultant & a member of the Board of Directors for Cardiff Oncology. S.A.H. reports receiving research funding from BMS/Celgene, and consulting fees from Janssen. K.G. reports consulting for Novartis. There are no conflicts of interest for other authors.

## Supporting information


Data S1.

## Data Availability

Researchers interested in the original data should contact the corresponding author or University of Nebraska Medical Center Investigator‐Initiated Trial Office at iitoffice@unmc.edu. A Data Use Agreement will be required.
