# Supplementary material for: A Phase II Trial of Geriatric Assessment‐Guided Selection of Treatment Intensity in Older Adults With AML
Source: Am J Hematol. 2025 Apr 29;100(7):1163–72. doi: 10.1002/ajh.27694 (PMC12146815; doi:10.1002/ajh.27694)
Supplement: Supplementary file 1 — Data S1. [file AJH-100-1163-s001.pdf]

Supplementary Table 1. AML subtypes based on 2016 WHO classification

**AML subtypes**	Number (%)
**AML with recurrent genetic abnormalities**	
Core binding factor AML	2 (2.7%)
AML with mutated NPM1	14 (19.2%)
AML with biallelic CEBPA mutation	2 (2.7%)
AML with mutated RUNX1	13 (17.8%)
**AML with myelodysplasia-related changes**	13 (17.8%)
**Therapy-related myeloid neoplasms**	16 (22.0%)
**AML, not-otherwise specified**	11 (15.1%)
**Myeloid sarcoma**	2 (2.7%)

WHO World Health Organization

Supplemental Table 2: Characteristics and Outcomes of Older Adults with Good-risk Acute Myeloid Leukemia

HCT CI	Katz ADL Score	Lawton IADL Score	SPPB	MoCA Score	Cytogenetic results	Positive mutation studies	Chemotherapy	Best response	Survival in months
**5**	**5**	8	**4**	**19**	46,XX[20]	NPM1, SF3B1, IDH1, DNMT3A	Decitabine	Resistant disease	2
2	**5**	**5**	10	**23**	46,XX[20]	NPM1*	Decitabine	Complete remission	Beyond 24
**7**	6	**7**	10	**19**	46,XY[20]	**Biallelic CEPBA**, TET2, SRSF2	Decitabine	Not evaluated before adding lenalidomide to decitabine but achieved complete remission afterwards	24
2	**5**	8	**1**	**24**	46,XY[21]	NPM1, KIT, TET2, ASXL1, SH2B3, MPL	Azacitidine, venetoclax	Resistant disease	6
**5**	**5**	8	9	29	46,XX[20]	NPM1, SRSF2, STAG2	Azacitidine, venetoclax	Complete remission	Beyond 24
**4**	**3**	**2**	**0**	**18**	46,XX,**inv(16)(**p13.1q22)[15]/ 46,XX[5]	No FLT3 ITD or KIT mutations	Decitabine (venetoclax was added with the second cycle)┼	Complete remission with incomplete blood count recovery	12
**4**	6	8	**4**	**24**	47,XX,+8[20]	FLT3-TKD, NPM1	Azacitidine, venetoclax	Complete remission	6
**8‡**	6	8	**8**	**22**	47,XX,+8,**inv(16)(**p13.1q22), inv(22)(q11.2q13.3)[20]	NRAS, WT1	Azacitidine, venetoclax	Complete remission	6 (developed stroke after first cycle in the setting of atrial fibrillation)
**5**	6	8	**0**	**21**	47,XY,+8[3]/46,XY[17]	KRAS, U2AF1, NPM1, TET2	Decitabine, venetoclax	Resistant disease	7
**3**	6	8	**2**	**23**	46,XY[20]	IDH1, NRAS, NPM1, DNMT3A	Decitabine, venetoclax	Complete remission	Beyond 24
2	6	8	**6**	**24**	45,X,-Y[16]/46,XY[4]	NPM1, TET2, MPL	Azacitidine, venetoclax	Complete remission	Beyond 24
**5**	**5**	8	**2**	26	46,XX[20]	NPM1, KRAS, NRAS, TET2	Azacitidine, venetoclax	Complete remission	Beyond 24

0§	6	8	**9**	29	46,XX[20]	NPM1, FLT3 TKD, IDH2, DNMT3A, 2 PTPN11 mutations	Decitabine, venetoclax	Complete remission	Beyond 24

ADL Activities of Daily Living, HCT CI Hematopoietic Cell Transplantation Comorbidity Index, IADL Instrumental ADL, MoCA Montreal Cognitive Assessment, SPPB Short Physical Performance Battery

Scores in bold indicate impairments based on the pre-defined cutoffs. All the patients with exception of the last patient had impairment in 2 or more domains.

*A limited myeloid mutation panel was performed and was negative for FLT3ITD, IDH1, IDH2 and CEBPA mutation. Other mutations were not tested.

┼The treating physician felt the patient was not fit for either intensive chemotherapy such as 7+3 or post-remission consolidation with intermediate-dose cytarabine.

‡Had multiple comorbidities including prior kidney transplant, chronic kidney disease with elevated creatinine, carotid artery stenosis, coronary artery disease, diabetes, hypertension, systemic lupus erythematosus. Felt to have treatment-related AML because of prior exposure to azathioprine.

§Whereas HCT CI was considered to be 0, this 70-year old patient had a history of hypertension, significant smoking, and diabetes previously treated with insulin but off insulin following weight loss; hemoglobin A1c of 8.2 after the diagnosis of AML and preceding albuminuria demonstrated poor diabetes control since coming off insulin.

Supplementary Table 3. Baseline comorbidities in the study population, captured by Hematopoietic Cell Transplantation Comorbidity Index

Individual Comorbidity	Count	Percent	Number of Comorbidities	Count	Percent
Depression or anxiety	21	28.8	0	15	20.5%
Prior solid malignancy	20	27.4	1	16	21.9%
Diabetes	18	24.7	2	17	23.3%
Cardiovascular	18	24.7	3	14	19.2%
Obesity	14	19.2	4	7	9.6%
Pulmonary, moderate (n=9) or severe (n=2)	11	15.1	5	4	5.5%
Infection	9	12.3			
Arrhythmia	7	9.6			
Hepatic mild (n=3) or moderate-severe (n=3)	6	8.2			
Renal	5	6.8			
Rheumatologic	3	4.1			
Other*	8	10.9			

*Other comorbidities included inflammatory bowel disease (n=2), heart valve disease (n=3), cerebrovascular disease (n=2), and peptic ulcer (n=1).

Supplementary Table 4. Number of the trial participants meeting unfit criteria by individual geriatric domains. X denotes assessment scored as impaired.

Number of vulnerable criteria met	N (%)	Pattern Count	HCTCI	Katz ADL	Lawton ADL	SPPB	MOCA
0	8 (11)	8					
1	11 (15)	2					X
		3				X	
		1			X		
		5	X				
2	15 (21)	9				X	X
		1		X		X	
		3	X				X
		2	X			X	
3	25 (34)	2			X	X	X
		3		X		X	X
		1		X	X		X
		11	X			X	X
		2	X		X		X
		1	X		X	X	
		5	X	X		X	
4	11 (15)	3		X	X	X	X
		3	X		X	X	X
		5	X	X		X	X
5	3 (4)	3	X	X	X	X	X

ADL activities of daily living, HCT CI Hematopoietic Cell Transplantation Comorbidity Index, IADL instrumental ADL, MOCA Montreal Cognitive Assessment, N number of patients, SPPB Short Physical Performance Battery

Supplementary Table 5. Geriatric assessment results and outcomes based on fitness at baseline

Domain	Fit<br>(n = 8)	Unfit<br>(n = 65)
**Assessments that were used to determine fitness for intensive chemotherapy**		
**Comorbidity burden**		
Hematopoietic Cell Transplantation Comorbidity Index (HCT CI)	0(0-0.5)	3(2-5)
**Physical function**		
Katz ADL Score	6(6-6)	6(5-6)
Lawton ADL Index Score	8(8-8)	8(8-8)
Total Score Short Physical Performance Battery	11(10-11)	7(3-9)
**Cognitive screen**		
MOCA score	28 (26-29)	23 (21-24)
**Other scores that were not used to determine fitness for intensive chemotherapy**		
Patient Health Questionnaire-9 Score	4(2-6)	6(3-11)
Body Mass Index	27(25.5-29.5)	28(24-34)
Mini Nutritional Assessment (MNA) Score	12.5(11.5-14)	11(8-12)
KPS Score	90(80-90)	80(70-80)
Social support	95 (86-95)	91 (81-95)
Weight Loss in the last 3 months (kilograms)	0 (0-0)	1.35 (0-3.25)
Falls in last 6 months (Yes)	0 (0%)	10 (15.4%)
History of Dementia or Delirium (Yes)	0 (0%)	7 (10.8%)
History of Urinary Incontinence (Yes)	1 (12.5%)*	12 (18.5%)
History of Stool Incontinence (Yes)**	0 (0%)	5 (7.9%)
**Mortality and remission results** (intent-to-treat analysis)		
**30-day Mortality**	0 (0%)	5 (7.7%)
**90-day Mortality**	1 (12.5%)	15 (23.1%)
**One-year survival (Kaplan-Meier Estimates with 95% confidence interval)**	42.9%<br>(18.2-100%)	46.3%<br>(35.5-60.5%)
**Complete remission as best response**	3 (37.5%)	26 (40.0%)

**Complete remission with or without blood count recovery as best response**	4 (50.0%)	34 (52.3%)

Scores for geriatric assessment results are presented as median (inter-quartile range).

*This patient had a history of urine incontinence in the past but had full bladder control in the more recent time.
**One fit and two unfit patients had missing data.

Supplementary Table 6. Best Response Rates based on AML risk categories

**Best Response**	**AML Risk Category**		
	**Good** (n = 13)	**Intermediate** (n = 16)	**Adverse** (n = 44)
**Complete remission**	8 (61.5%)	8 (50.0%)	13 (29.6%)
**Complete remission with incomplete blood count recovery**	1 (7.7%)	1 (6.3%)	7 (15.9%)
**Morphologic leukemia free state**	0 (0%)	0 (0%)	2 (4.5%)
**Non-evaluable**	1 (7.7%)	1 (6.3%)	5 (11.4%)
**Resistant disease**	3 (23.1%)	6 (37.5%)	17 (38.6%)

Supplementary Table 7. Causes of death

Cause of death	Fit patients, N(%)	Unfit patients, N(%)
Acute myeloid leukemia	2 (40%)	21 (51%)
Infection	2 (40%)	5 (12%)
Respiratory failure	0	3 (7%)
Miscellaneous or unknown	1 (aGVHD) (20%)	12 (30%)*
Total deaths	5 (100%)	41 (100%)

aGVHD acute graft-versus-host disease

*This included lost to follow-up prior to death (n=4), bone marrow aplasia post-chemotherapy (n=2), transition to hospice (n=2), aGVHD (n=2), anoxic brain injury (n=1), subdural hematoma (n=1)

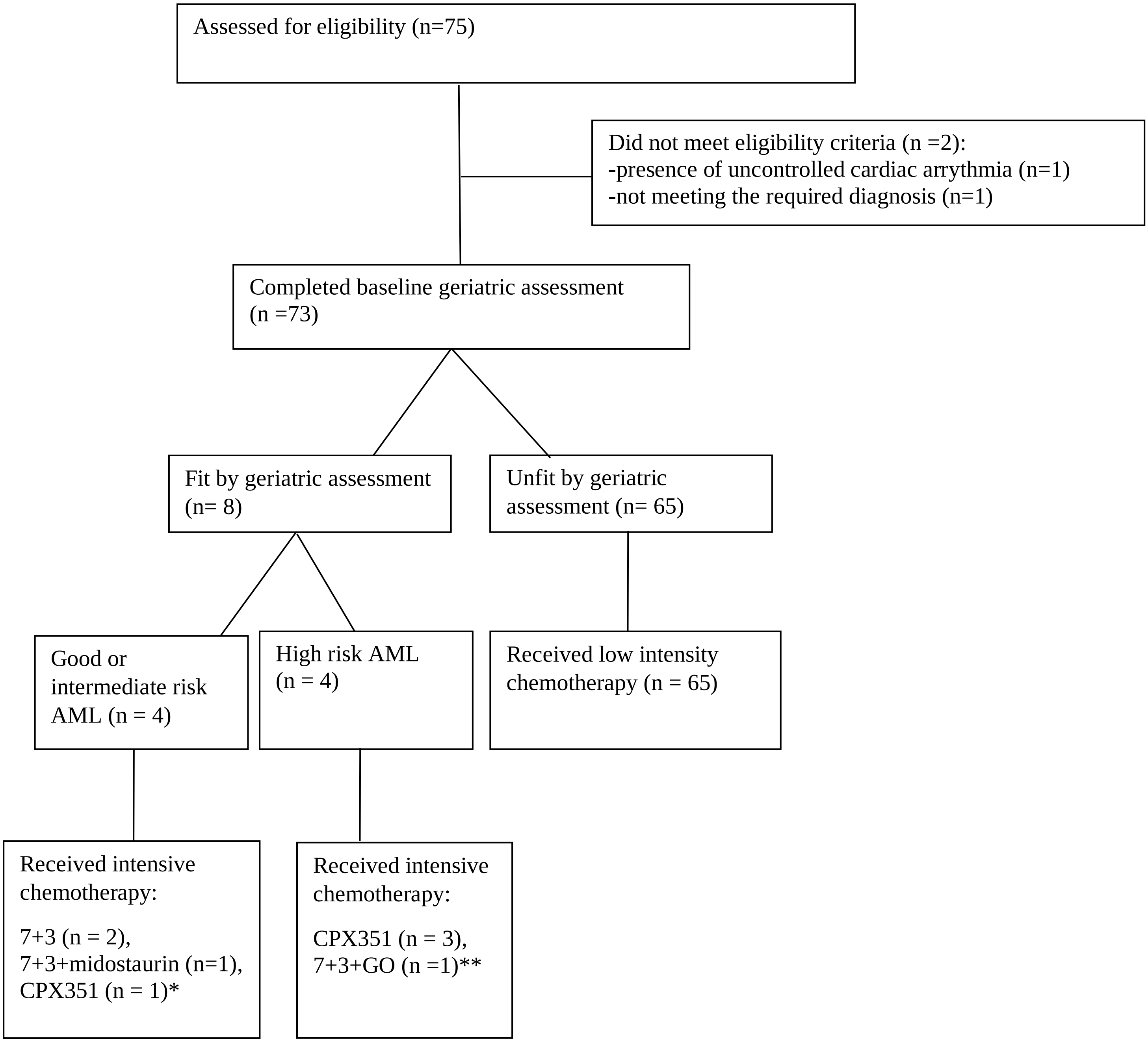

* One patient with 46,XX,t(1;3)(p36.3;q21)[20] was considered to have intermediate-risk AML per the 2017 ELN risk classification and AML MRC per the 2016 WHO classification, hence received CPX351.

**The patient had intermediate risk disease by cytogenetics and received 7+3+gemtuzumab ozogamicin (GO) per protocol but subsequently was found to have high-risk mutations (ASXL1, SRSF2).

Supplemental Figure 1. CONSORT diagram showing patients' disposition.

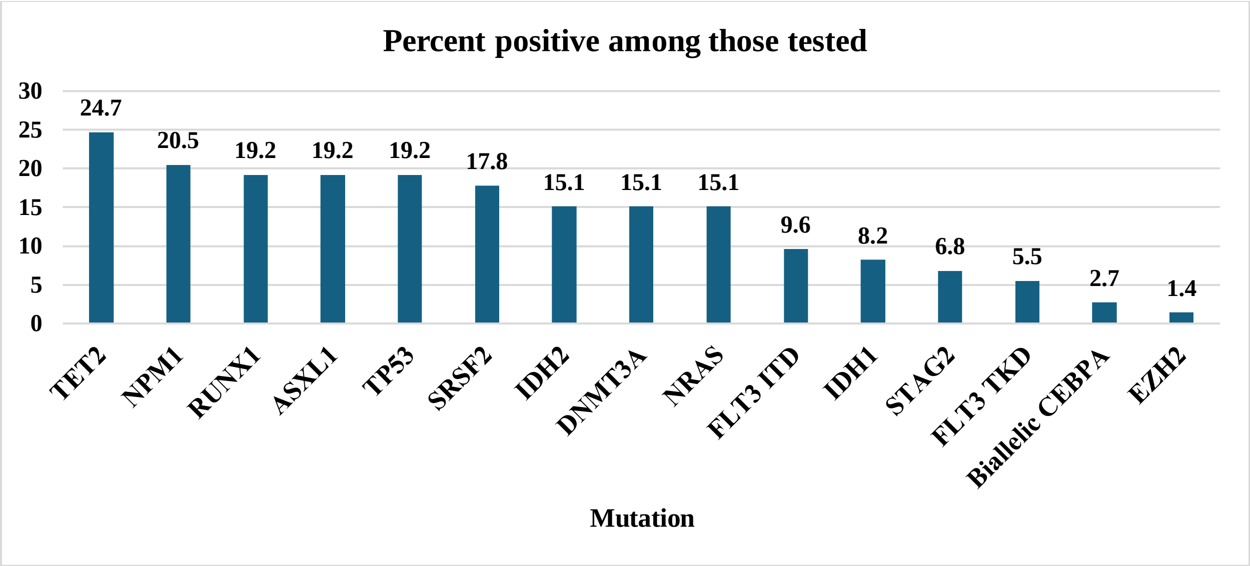

Supplemental Figure 2. Frequency of various mutations. Twelve patients did not undergo multi-gene mutation panel testing. Seven out of these 12 patients had limited testing such as testing of FLT3 ITD, NPM1, IDH1 or IDH2 mutations.

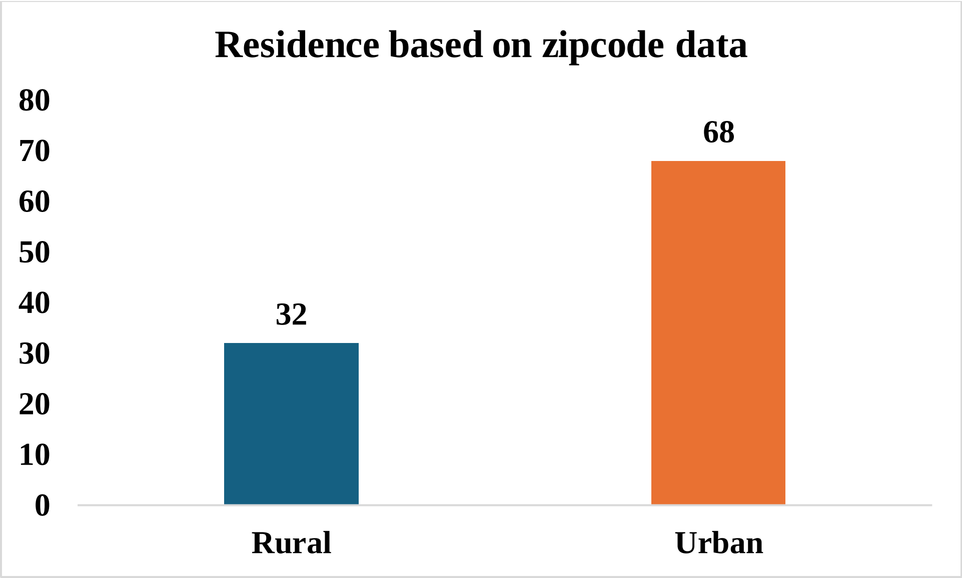

Supplemental Figure 3a. Residence based on zipcode data. Twenty-three patients resided in rural areas, and fifty patients resided in urban areas. This was determined by entering patient's zip code into the 2022 Census Reporter to obtain population size. The population size at each zip code was used to determine urban versus rural based on the USDA Economic Research Service definition of rural towns populating fewer than 5,000 people.

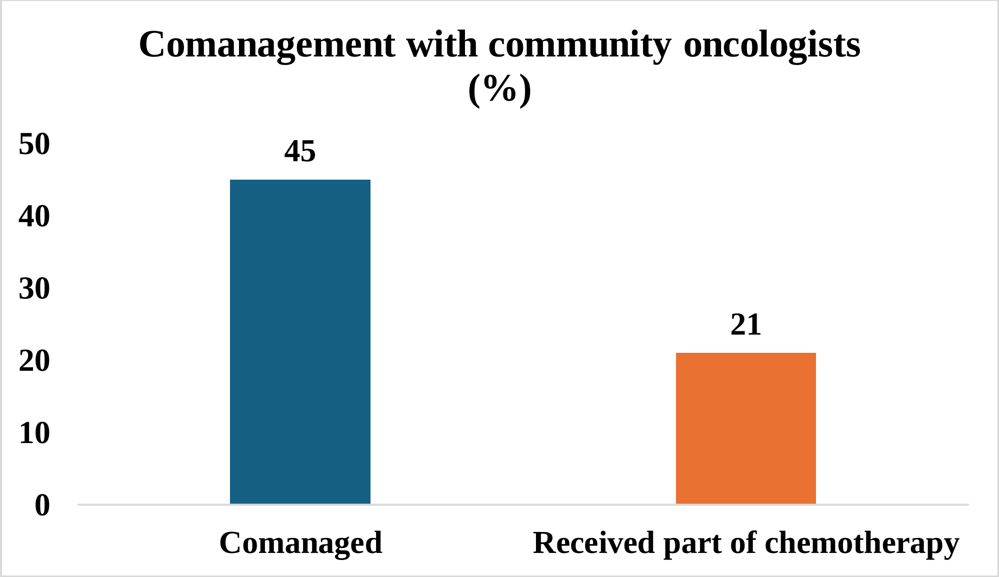

Supplemental Figure 3b. Comanagement with community oncologists. Thirty-three patients were comanaged with community oncologists, and fifteen received part of chemotherapy in community cancer centers.

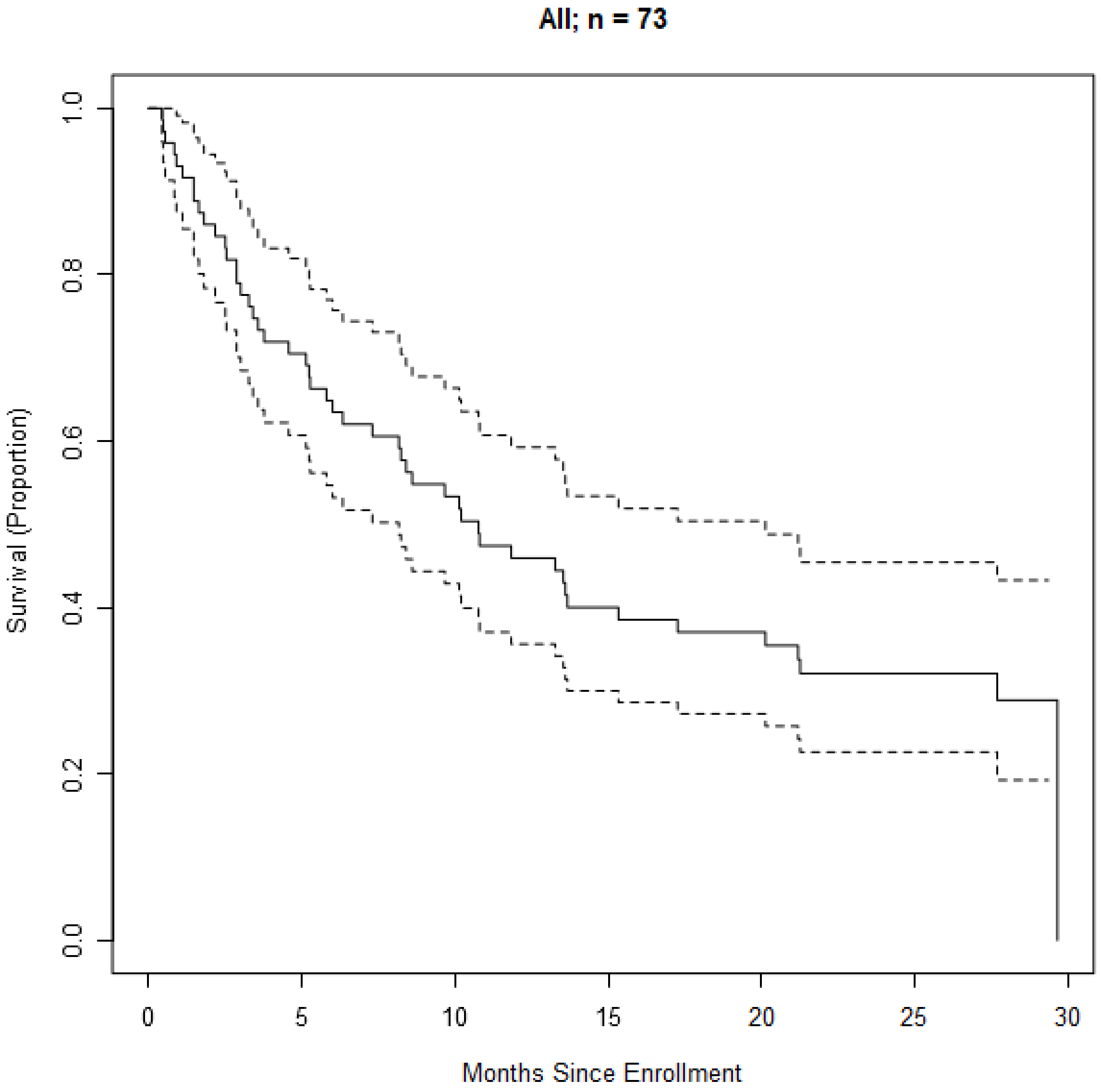

Supplemental Figure 4a. Survival of the entire study participants

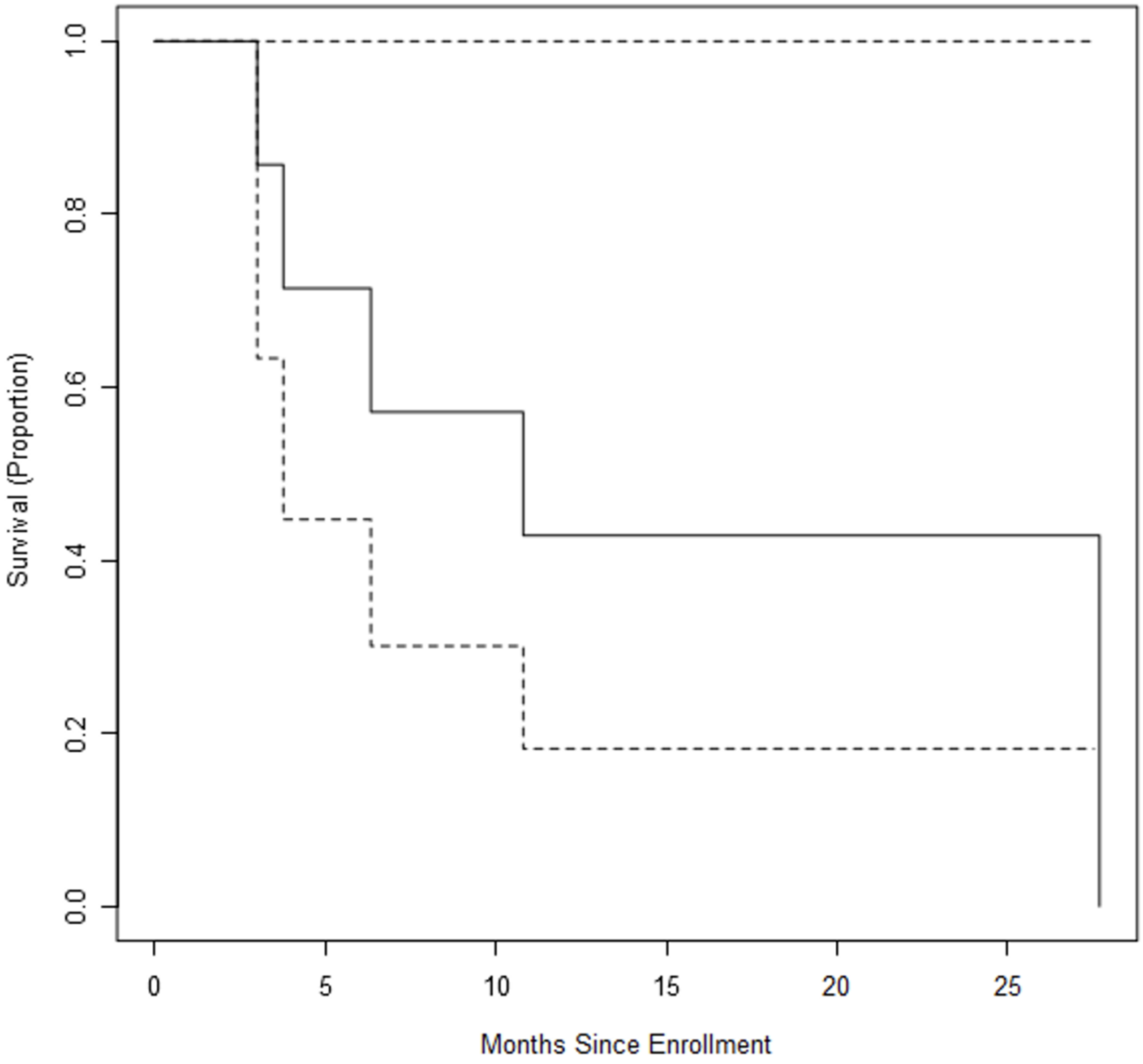

Figure 4b. Survival of the study participants, who were deemed fit (n=8).

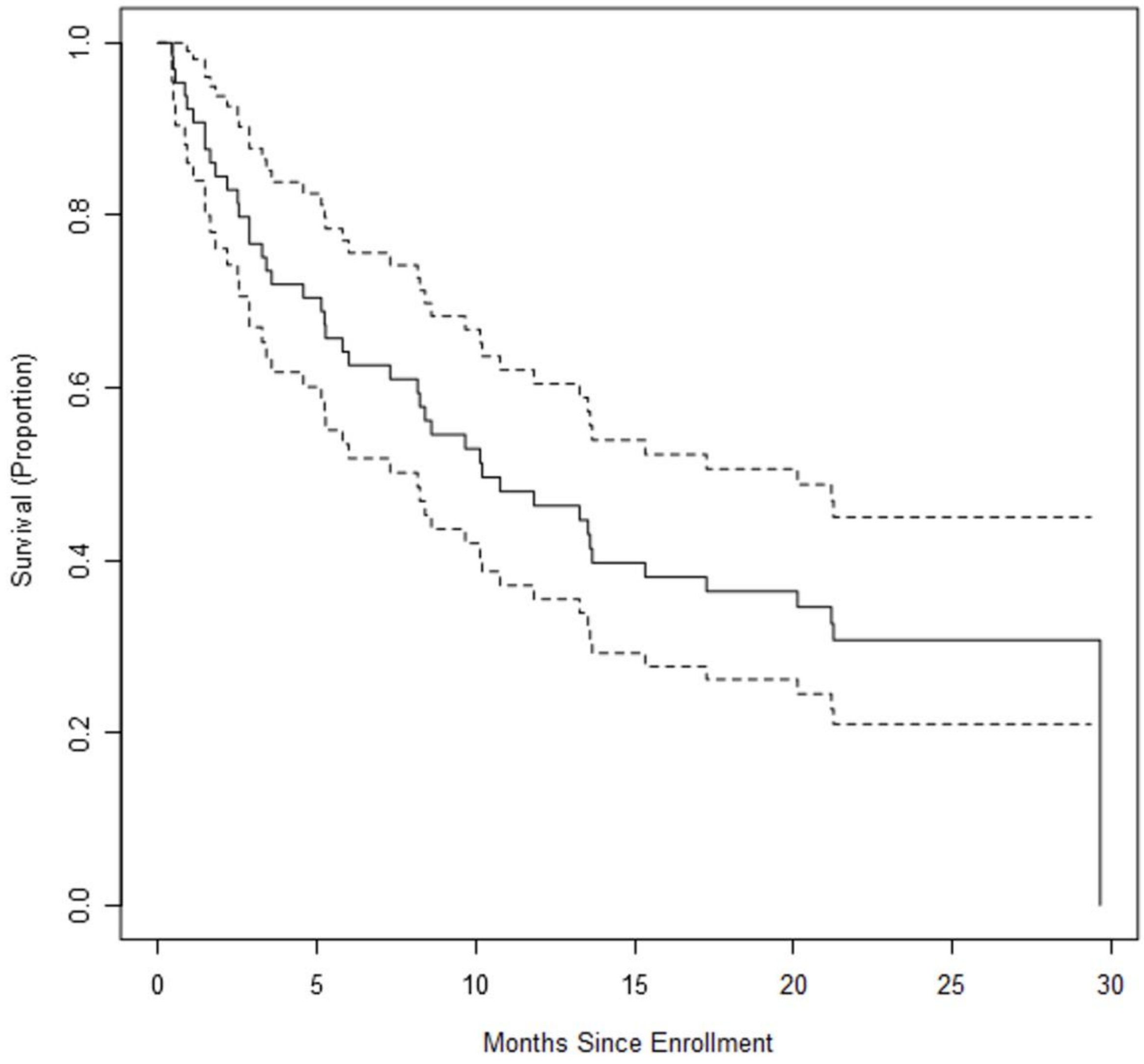

Figure 4c. Survival of the study participants, who were deemed unfit (n=65).
